# Granulomatosis with Polyangiitis Presenting as Pyrexia of Unknown Origin, Leukocytosis, and Microangiopathic Haemolytic Anemia

**DOI:** 10.1155/2017/6484092

**Published:** 2017-07-24

**Authors:** Sima Terebelo, Iona Chen

**Affiliations:** Maimonides Medical Center, Brooklyn, NY, USA

## Abstract

A 66-year-old woman presented to the Emergency Department with a florid sepsis-like picture, a two-week history of fever, relative hypotension with end organ ischemia (unexplained liver enzyme and troponin elevations), and nonspecific constitutional symptoms. She was initially found to have a urinary tract infection but, despite appropriate treatment, her fever persisted and her white blood cell count continued to rise. During her hospitalization the patient manifested leukocytosis to 47,000 WBC/*μ*L, ESR 67 mm/hr (normal range 0–42 mm/hr), CRP 17.5 mg/dL (normal range 0.02–1.20 mg/dL), and microangiopathic haemolytic anemia, with declining haemoglobin and haematocrit. An infectious aetiology was not found despite extensive bacteriologic studies and radiographic imaging. The patient progressed to acute kidney injury with “active” urinary sediment and proteinuria. Kidney biopsy results and serological titres of myeloperoxidase positive perinuclear-antineutrophil cytoplasmic antibodies (MPO+ p-ANCA) led to a diagnosis of granulomatosis with polyangiitis. Immunosuppressive treatment with high dose methylprednisolone and rituximab led to resolution of the leukocytosis and return of the haemoglobin and haematocrit values toward normal without further signs of hemolysis.

## 1. Introduction

Granulomatosis with polyangiitis (GPA) is an uncommon autoimmune disease characterized by a pauci-immune necrotizing vasculitis of small and medium sized vessels. It most commonly occurs in Caucasian patients between 45 and 65 years, without gender predilection, and characteristically affects the upper and lower respiratory tract and kidneys. We report a 66-year-old Afro-Caribbean woman whose presenting symptoms and findings suggested sepsis syndrome and included fever, hypotension with end organ ischemia, leukocytosis to 47,000 WBC/*μ*L, and microangiopathic haemolytic anemia (MAHA). An extensive workup failed to show any infectious or neoplastic aetiology. The patient was ultimately diagnosed with GPA based on kidney biopsy results and MPO+ p-ANCA. To our knowledge, leukocytosis to this extent has not been previously described with GPA. Additionally, MAHA rarely accompanies GPA.

## 2. Description of Patient

A 66-year-old Afro-Caribbean woman presented to the Emergency Department with complaints of weakness, fever, fatigue, myalgia, hypotension × 2 weeks, and increased urinary frequency. The patient has a past medical history of hypertension, hypothyroidism, hearing loss, tinnitus, and “benign pulmonary nodules.” Two weeks prior to presentation, the patient began experiencing daily fevers of 102 degrees Fahrenheit (38.9 degrees Celsius), severe body aches, nonproductive cough with pleuritic chest pain, and low blood pressure (90–100 mmHg systolic) without ingestion of antihypertensive medications. She visited her primary care physician three days prior to presentation and was given amoxicillin for presumed upper respiratory infection; however her symptoms continued to worsen.

The patient disclosed a two-year history of intermittent episodes of bronchitis and haemoptysis, which were treated with multiple courses of oral antibiotics. She had presented to our institution twice for evaluation of haemoptysis. On the first occasion, 1 year ago, she had minimal bloody expectorant and was treated for bronchitis. On her second presentation, three months ago, frank haemoptysis was present and the patient was admitted. Sputum samples were negative for acid fast bacilli and cultures were negative for tuberculosis. The patient was ultimately treated for community acquired pneumonia. Pulmonary nodules noted on radiographic imaging had been evaluated in an outpatient setting, and the patient indicated that she had been informed that these were “benign.”

The patient was a nonsmoker and did not consume alcohol. She worked as a patient care technician in a hospital and had a history of positive purified protein derivative skin test for tuberculosis (PPD+). She recently traveled to Haiti.

In the Emergency Department, the patient was afebrile (had just taken paracetamol), with respiratory rate = 23, blood pressure 114/67, white blood cell count (WBC) 24,000/*μ*L, 87% neutrophils, 4% lymphocytes, 7% monocytes, haemoglobin 9.1 g/dL with +anisocytosis and target cells, platelets 513,000/*μ*L, troponin 0.11 ng/mL (normal 0.00–0.04 ng/mL), AST 399 IU/L (normal 8–26 IU/L), ALT 368 IU/L (normal 6–51 IU/L), alkaline phosphatase 131 IU/L (normal 33–92 IU/L), total bilirubin 0.4 mg/dL (normal 0.4–1.1 mg/dL), 0.1 mg/dL direct bilirubin (normal 0.1–0.2 mg/dL), and albumin 2.5 g/dL (normal 3.6–4.6 g/dL).

Physical exam was notable for decreased breath sounds over the right lower lobe and minimal lower extremity oedema bilaterally. There were no rashes, swollen joints, digital ulcers, or loss of digit pulp.

### 2.1. Clinical Course and Diagnostic Assessment

Initial workup was significant for a urinary tract infection. Despite appropriate antibiotic therapy the patient continued to be intermittently febrile accompanied by increasing leukocytosis, with neutrophil counts ranging from 80 to 86%. Repeat urine culture was negative. No source of infection was found despite extensive investigation. Multiple blood cultures were negative. CT chest demonstrated chronic scarring and architectural distortion of the right upper lobe, thought to be due to prior granulomatous disease, which was unchanged from a prior CT. A left lung nodule was noted, unchanged from the patient's previous CT on 10/2015. Sputum samples were negative for acid fast bacilli by fluorochrome methodology. Further diagnostic studies were initiated due to the persistence of fever. Echocardiogram was negative for valvular vegetation. CT scan of the abdomen and pelvis was negative for occult abscess or osteomyelitis. MRI of the abdomen and pelvis was negative for infectious processes and pelvic ultrasound did not reveal gynecologic pathology. A peripheral blood smear examined by a haematology/oncology consultant was interpreted as reactive, without signs of neoplasia, and therefore there was no indication for bone marrow biopsy.

**Figure 1:**
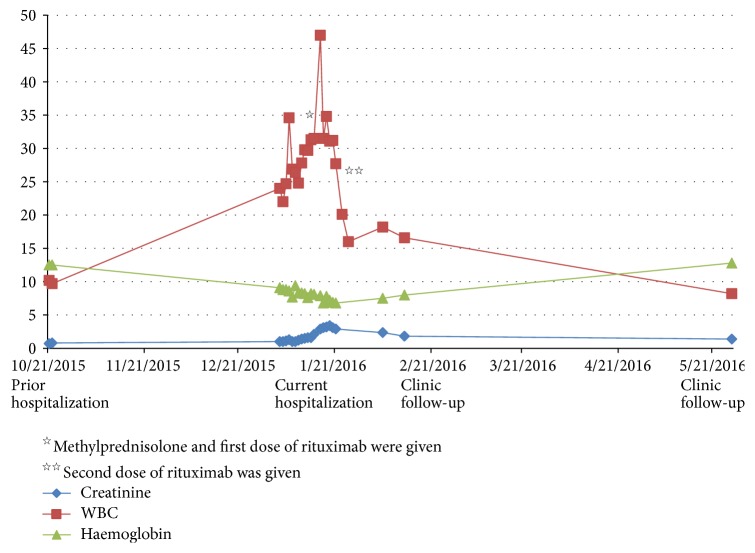
*Relationship between serum creatinine, WBC, and haemoglobin*. Upon initial presentation to our hospital several months earlier, patient had normal laboratory values. At this admission the patient had leukocytosis, anemia, and rising creatinine values. Administration of methylprednisolone and rituximab (star) was associated with an initial rise in WBC count. Within several days leukocyte counts began to decline, most notably after the second rituximab infusion (two stars). Haemoglobin and creatinine levels also responded appropriately. At the outpatient clinic follow-up the patient received another two doses of rituximab. Laboratory testing continued to show improved WBC count, haemoglobin, and creatinine levels.

WBC count continued to increase from the admission level of 24,000/*μ*L to a peak of 47,000/*μ*L. Haemoglobin and haematocrit values gradually declined from admission values of 9.1 g/dL and 29.9% to 6.6 g/dL and 20% (Figure 1); schistocytes and target cells were identified on peripheral smear. Coombs test was negative for direct and indirect antibodies. LDH rose from 251 IU/L to 409 IU/L (normal 84–193 IU/L) and haptoglobin was <3 (normal 34–200 mg/dL), consistent with microangiopathic haemolytic anemia (MAHA). Other relevant laboratory findings included serum iron 14 mcg/dL (normal 64–196 mg/dL), transferrin 105 mg/dL (normal 192–321 mg/dL), TIBC 147 mcg/dL (normal 279–449 mcg/dL), ferritin 452 ng/mL (normal 4.8–94.4 ng/mL), and reticulocyte count 5.6%, with absolute reticulocytes 0.160, and corrected reticulocyte count 2.9%.

Liver enzymes and cardiac troponin levels decreased to normal. Serological testing was negative for acute or chronic viral hepatitis, Epstein Barr virus, or cytomegalovirus infections.

Creatinine gradually increased from baseline 0.9 mg/dL (normal 0.5–1.2 mg/dL) to peak of 3.4 mg/dL. Urine microscopy revealed 5–10 red cell casts/LPF and 2–5 coarse granular casts/LPF. Urine protein : creatinine ratio was 1.8-gram protein/gram creatinine (normal <0.16 g/g creatinine) and increased to 2.4-gram protein/gram creatinine. Markers of inflammation revealed ESR 67 mm/hr (normal 0–42 mm/hr) and CRP 17.5 mg/dL (normal 0.02–1.20 mg/dL). Inflammatory markers were only measured once during the patient's hospital course. ANA was weakly positive 1 : 80 (homogenous) and p-ANCA was positive, 1 : 160, with MPO 115.2 units (normal ≤ 20.0 units). Pertinent negative laboratory values included c-ANCA negative, PR3 negative, C3 113 mg/dL (normal 75–161 mg/dL), C4 22 mg/dL (normal 14–45 mg/dL), Rheumatoid Factor negative, antiglomerular basement membrane antibody negative (<0.02), and urine eosinophil smear negative.

**Figure 2:**
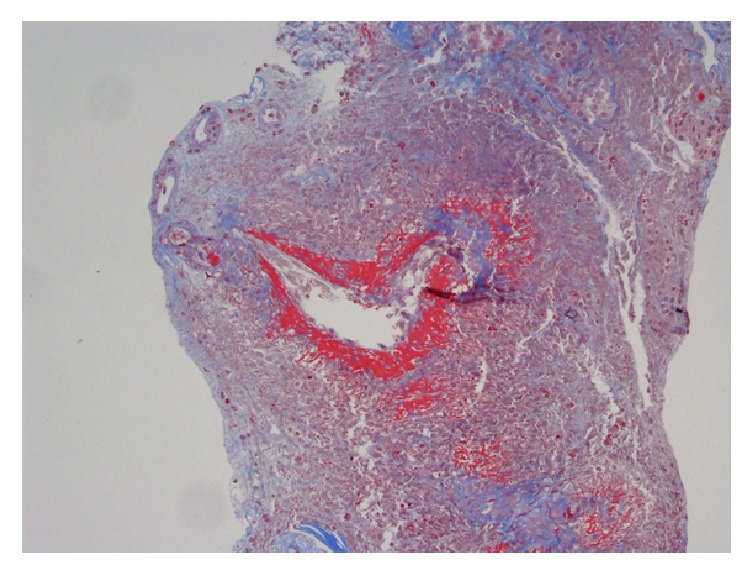
Full thickness fibrinoid necrosis of the vessels with surrounding interstitial necrosis.

**Figure 3:**
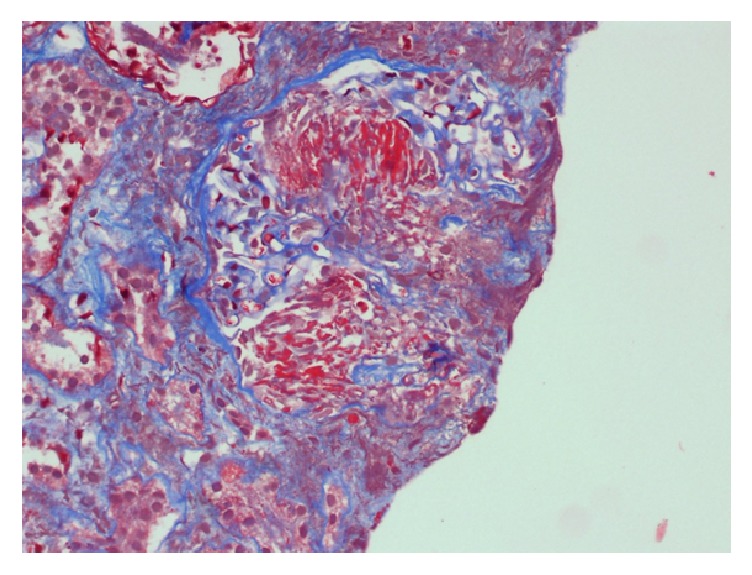
Tubular atrophy with dense lymphocytic inflammatory infiltrate and interstitial fibrosis.

**Figure 4:**
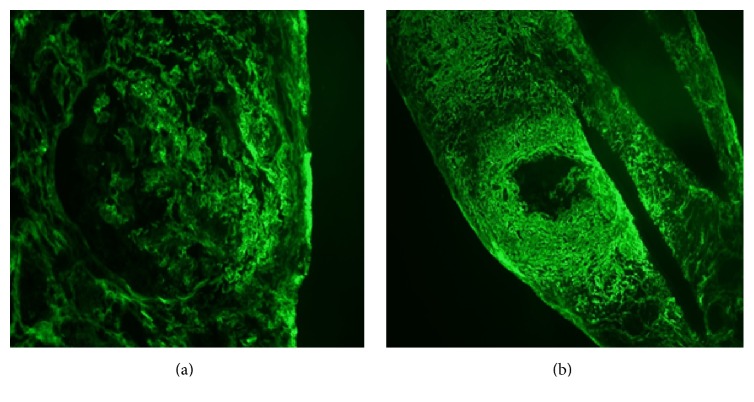
Fibrinogen staining shows cellular crescents (a) and necrosis of an artery (b).

Renal biopsy demonstrated pauci-immune necrotizing glomerulonephritis with crescents and vascular and interstitial necrosis. There was full thickness fibrinoid necrosis of the vessels with surrounding interstitial necrosis. There was 30% interstitial fibrosis associated with tubular atrophy and dense lymphocytic inflammatory infiltrate. Fibrinogen staining showed crescents and necrosis of an artery (Figures 2–4).

### 2.2. Treatment/Outcome

The patient was treated with methylprednisolone 1000 mg IV × 5 days. Rituximab 375 mg/m^2^ was started while being inpatient and was continued weekly for a total of 4 weeks. The patient had been manifesting frequent fevers within the range of 100.4–102.7 degrees Fahrenheit (38.0–39.3 degrees Celsius). The day following solumedrol infusion fevers ceased and patient's body temperature thereafter remained in the normal range. WBC count initially rose from 35,000/*μ*L with 87% neutrophils to 47,000/*μ*L with 82% neutrophils after methylprednisolone administration for two days and after the first dose of rituximab was administered. The following day WBC count declined to 31,500/*μ*L with 83% neutrophils and continued to decline gradually. One week after beginning methylprednisolone and rituximab therapy WBC count was 25,700/*μ*L with 81% neutrophils and declined to 20,100/*μ*L with 86% neutrophils following the second dose of methylprednisolone. Creatinine slowly declined and haemoglobin and haematocrit levels slowly rose. Urine protein : creatinine ratio declined to 1.6-gram protein/gram creatinine. Corticosteroids were gradually tapered. The patient tolerated the treatment well and clinical symptomatology resolved. Lab values, 5 months after hospitalization, revealed WBC 8.2k/*μ*L, haemoglobin 12.8 g/dL, haematocrit 39.2%, platelets 210k/UL, BUN 22 mg/dL and creatinine 1.4 mg/dL, AST 23 IU/L, and ALT 16 IU/L (Figure 1).

## 3. Discussion and Literature Review

Over a 2-year period, the patient experienced limited lung disease and developed hearing loss and tinnitus. She then abruptly developed a sepsis-like condition, which rapidly progressed to include persistent fever, increasing leukocytosis, MAHA, and acute necrotizing pauci-immune glomerulonephritis. The lack of response to antibiotic therapy prompted a search for occult infection or malignancy. Despite multiple blood and sputum cultures, extensive imaging studies (including a CT of the chest, abdomen, and pelvis, an MRI of the abdomen and pelvis, and a pelvic ultrasound), and peripheral blood smear evaluation by an oncologist, no source of abscess, infection, or neoplasm could be found to explain the patient's fevers and leukocytosis. The increase in creatinine and onset of proteinuria with active urinary sediment led to renal biopsy, the histology of which supported the diagnosis of GPA in the setting of the past history of haemoptysis, hearing loss, tinnitus, and positive serological markers for p-ANCA and MPO.

GPA is an uncommon autoimmune disease most prevalent in Caucasian patients with disease onset usually between 45 and 65 y [1]. Notably, the prevalence of GPA is very low in non-Caucasians, and this patient is Afro-Caribbean. In a survey of 701 patients in North America, only 2% of patients diagnosed with GPA were not Caucasian [1]. The diagnosis of GPA can be challenging, as the entity commonly presents with nonspecific symptoms such as fatigue, joint pains, and sinusitis [1–3]. Organ systems involved at the time of diagnosis are the upper and lower airways, ears, lung, joints, and kidneys [1–3]. Most patients are diagnosed within 3–12 months from the onset of symptomatology, and on average two organ systems are involved at time of diagnosis [1].

Our patient had a two-year indolent phase with minor exacerbations prior to her acute presentation. Isolated organ system involvement, such as isolated lung nodules, is rare in GPA. The French Vasculitis Study Group identified 16 patients with isolated GPA occurring for as long as 58 months without progression to systemic disease [4]. However, such patients may progress if followed long enough. One case report described a patient with limited tracheobronchial disease which then suddenly flared twenty years after the initial presentation [5].

Unexplained fever and increasing leukocytosis were significant features of our patient's presentation and course. In one retrospective survey, fever was described as the first symptom in 33% of patients [1]. Fever suggestive of an infectious aetiology is not uncommon in GPA. Several case reports describe fever and pulmonary symptoms with abnormal chest imaging studies (often initially interpreted to be pneumonia), as in our patient's diagnosis from her prior admission [6–8]. Tuberculosis was suspected in our patient on both hospitalizations due to exposure risks in Haiti and in her role as a healthcare worker, along with the presence of a positive tuberculin skin test.

Occasionally GPA can present with protracted fever without localizing signs, as in our patient, although this is uncommon. Pyrexia of unknown origin (PUO) is most commonly secondary to infectious disease. Fever secondary to occult collagen vascular disease is less common [9, 10]. In a retrospective cohort of 857 patients, 16% (137 patients) had fever due to collagen vascular disease. Only 3/137 (0.3% of the entire cohort) had fever secondary to GPA [11]. Our patient's fever resolved completely after the first dose of methylprednisolone.

Our patient's course was unusual in that her WBC count increased to 47,000. As such, there was significant concern for infectious or neoplastic source. We were unable to find an infectious source despite extensive microbiologic studies and imaging. The decision to begin treatment with high dose corticosteroids remained difficult in face of the florid leukocytosis, despite the negative sepsis workup. To our knowledge, leukocytosis at this level has not been previously described in the literature in association with GPA. The leukocytosis resolved completely after treatment with rituximab, further supporting its association with GPA.

Neutrophilic leukocytosis is commonly seen in association with an acute infectious process [12]. At times inflammatory processes or physiologic stressors can stimulate leukocytosis. For example, patients with rheumatoid arthritis, adult Still's disease, and noncystic fibrosis bronchiectasis have been found to develop leukocytosis with disease flare-ups [13–15]. Trauma patients have also been observed to have sterile neutrophilia with negative blood cultures [16]. In our patient, a possible mechanism for the observed leukocytosis could be as a response to extreme physiologic stress causing a profound inflammatory response and upregulating the immune system. Additionally, stimulation of bone marrow, such as that seen in haemolytic anemia, can rarely precipitate significant leukocytosis, possibly via overstimulation of the bone marrow in response to the anemia [17].

The patient's haemoglobin/haematocrit had declined in comparison to levels during the previous months. Her red blood cell levels continued to decline with schistocytes and target cells identified on peripheral smear. Laboratory evidence of MAHA included elevated LDH, undetectable haptoglobin, and negative Coombs test. MAHA is a rare complication of the immune activation in GPA and has been reported occasionally [18, 19]. In our patient the haemolytic anemia resolved completely after treatment with rituximab, further supporting the association.

Finally, c-ANCA with PR3 positive autoantibodies are diagnostic markers for GPA and are present in 70% to 90% of patients [20]. Our patient was p-ANCA MPO+, which is unusual in GPA, although it has been reported in 5% to 10% of cases [20]. In non-Caucasian populations, MPO+ p-ANCA may be more common. In a case series from China 60% of GPA patients were MPO+ p-ANCA. Those patients were more likely to have elevated serum creatinine at the onset of illness and less likely to have arthralgia, rash, and ophthalmic and ear involvement [20]. Our patient did not have arthralgia, rash, or ophthalmic involvement; however she did have tinnitus and a history of decreased hearing acuity.

## 4. Conclusion

GPA is a rare disorder with protean manifestations. It is important to consider the diagnosis of GPA early in order to begin immunosuppressive therapy. If not treated aggressively and promptly the patient can rapidly progress to renal failure. In this patient, the clinical picture of infection was misleading and made our decision to treat with corticosteroids and rituximab a very difficult one. We believe this is the first report of a patient with extreme leukocytosis in the setting of GPA and the absence of an infectious aetiology.

## References

[1] Abdou N. I., Kullman G. J., Hoffman G. S. (2002). Wegener's granulomatosis: survey of 701 patients in North America. changes in outcome in the 1990s. *The Journal of Rheumatology*.

[2] Takala J. H., Kautiainen H., Malmberg H., Leirisalo-Repo M. (2008). Wegener's granulomatosis in Finland in 1981-2000: clinical presentation and diagnostic delay. *Scandinavian Journal of Rheumatology*.

[3] Holle J. U., Gross W. L., Latza U. (2011). Improved outcome in 445 patients with Wegener's granulomatosis in a German vasculitis center over four decades. *Arthritis and Rheumatism*.

[4] Pagnoux C., Stubbe M., Lifermann F. (2011). Wegener's granulomatosis strictly and persistently localized to one organ is rare: Assessment of 16 patients from the French Vasculitis Study Group database. *Journal of Rheumatology*.

[5] Peters J. E., Salama A. D., Ind P. W. (2009). Wegener's granulomatosis presenting as acute systemic vasculitis following 20 years of limited tracheobronchial disease. *Journal of Laryngology and Otology*.

[6] Zubairi A. B. S., Liaquat H. B., Jusain S. J., Fatima K. (2009). Wegeners granulomatosis: a diagnostic challenge. *Journal of Pakistani Medical Association*.

[7] Spalding S. J., Cambria M., Arkachaisri T. (2009). Distinguishing wegener's granulomatosis from necrotizing community acquired pneumonia: a case report and comparison of radiographic findings. *Pediatric Pulmonology*.

[8] Paudyal B. P., Pantha S., Ranjitkar N., Manandhar A., Arjyal A. (2011). A diagnosis missed for several years-Wegener's granulomatosis. *Kathmandu University Medical Journal*.

[9] Islam M. A., Bagheri F., Bencomo D. (2007). Wegnener’s granulomatosis presenting as fever of unknown origin in an African-American male. *Proceedings of the Western Pharmacology Society*.

[10] Bayrak E., Dönderici Ö., Serter R., Efe F. K. (2010). A case with fever of unknown origin diagnosed as wegener granulomatosis. *Turkish Journal of Rheumatology*.

[11] Sipahi O. R., Senol S., Arsu G. (2007). Pooled analysis of 857 published adult fever of unknown origin cases in Turkey between 1990-2006. *Medical Science Monitor*.

[12] Lawrence Y. R., Raveh D., Rudensky B., Munter G. (2007). Extreme leukocytosis in the emergency department. *QJM*.

[13] Syed K. M., Pinals R. S. (1996). Leukocytosis in rheumatoid arthritis. *Journal of Clinical Rheumatology*.

[14] Pouchot J., Sampalis J. S., Beaudet F. (1991). Adult Stills disease: manifestations, disease course and outcomes in 62 patients. *Medicine*.

[15] Wilson C. B., Jones P. W., O'Leary C. J. (1998). Systemic markers of inflammation in stable bronchiectasis. *European Respiratory Journal*.

[16] Claridge J. A., Golob Jf J. F., Fadlalla A. M., Malangoni M. A., Blatnick J., Yowler C. J. (2009). Fever and leukocytosis in critically ill traua patients: it is not the blood. *Am Surg*.

[17] Jea S. J., Kim S. Y., Choi B. M., Lee J. H., Lee K. C., Woo C. W. (2007). A pediatric case of autoimmune hemolytic anemia followed by excessive thrombocytosis and leukocytosis. *Korean J Hematol*.

[18] Ross C. N., Reuter H., Scott D., Hamilton D. V. (1996). Microangiopathic haemolytic anaemia and systemic vasculitis. *British Journal of Rheumatology*.

[19] Jordan J., Manning M., Allen N. B. (1986). Multiple unusual manifestatiosn of Wegeners granulomatosis: breast mass, micrangiopathic haemolytic anemia, consumptive coagulopathy, and low erythrocyte sedimentation rate. *Arthritis Rheumatism*.

[20] Chen M., Yu F., Zhang Y., Zou W.-Z., Zhao M.-H., Wang H.-Y. (2005). Characteristics of Chinese patients with Wegener's granulomatosis with anti-myeloperoxidase autoantibodies. *Kidney International*.

